# Automating sleep stage classification using wireless, wearable sensors

**DOI:** 10.1038/s41746-019-0210-1

**Published:** 2019-12-20

**Authors:** Alexander J. Boe, Lori L. McGee Koch, Megan K. O’Brien, Nicholas Shawen, John A. Rogers, Richard L. Lieber, Kathryn J. Reid, Phyllis C. Zee, Arun Jayaraman

**Affiliations:** 1 Max Nader Lab for Rehabilitation Technologies and Outcomes Research, Shirley Ryan AbilityLab, Chicago, IL 60611 USA; 2 Department of Biomedical Engineering, Northwestern University, Evanston, IL 60208 USA; 3 Department of Neurology, Northwestern University, Chicago, IL 60611 USA; 4 Department of Physical Medicine and Rehabilitation, Northwestern University, Chicago, IL 60611 USA; 5 Medical Scientist Training Program, Northwestern University Feinberg School of Medicine, Chicago, IL 60611 USA; 6 Center for Bio-Integrated Electronics, Departments of Materials Science and Engineering, Biomedical Engineering, Electrical Engineering and Computer Science, Northwestern University, Evanston, IL 60208 USA; 7 Shirley Ryan AbilityLab, Chicago, IL 60611 USA

**Keywords:** Biotechnology, Biomarkers, Computational science

## Abstract

Polysomnography (PSG) is the current gold standard in high-resolution sleep monitoring; however, this method is obtrusive, expensive, and time-consuming. Conversely, commercially available wrist monitors such as ActiWatch can monitor sleep for multiple days and at low cost, but often overestimate sleep and cannot differentiate between sleep stages, such as rapid eye movement (REM) and non-REM. Wireless wearable sensors are a promising alternative for their portability and access to high-resolution data for customizable analytics. We present a multimodal sensor system measuring hand acceleration, electrocardiography, and distal skin temperature that outperforms the ActiWatch, detecting wake and sleep with a recall of 74.4% and 90.0%, respectively, as well as wake, non-REM, and REM with recall of 73.3%, 59.0%, and 56.0%, respectively. This approach will enable clinicians and researchers to more easily, accurately, and inexpensively assess long-term sleep patterns, diagnose sleep disorders, and monitor risk factors for disease in both laboratory and home settings.

## Introduction

Sleep is a complex physiological state influencing the homeostasis of brain function, autonomic nervous system (ANS) organization, and circadian rhythms.^1,2^ Sleep duration and temporal cycling of sleep stages, including rapid eye movement (REM) and various non-REM stages (NREM), heavily influence both objective and subjective sleep quality.^3^ Accurately mapping these elements of sleep architecture is crucial for identifying non-restorative sleep, diagnosing sleep disorders, and exposing symptoms that are related to cardiovascular, neurological, and psychosomatic conditions.^4,5^

At present, the gold standard technique for assessing sleep quality and state is laboratory polysomnography (PSG), which utilizes a combination of electroencephalography (EEG), electrocardiography (ECG), electrooculography (EOG), and electromyography (EMG) to identify sleep stages, wake/arousals, and ANS-based sleep changes. Laboratory PSG requires a dedicated physical space to conduct sleep assessments, as well as an on-site overnight staff to apply and monitor a plethora of wired and wireless physiological sensors and to evaluate the integrity of acquired data. Registered sleep technicians visually score PSG data post hoc in 30-s epochs to determine sleep stages.^6^ Critically, the financial costs and resource burden associated with PSG data acquisition, the subsequent scoring of sleep records, and the discomfort to patients can outweigh the benefit of this system’s high accuracy^7^ and limit its potential for long-term sleep assessment.

Wrist actigraphy (WA) is traditionally used to assess long-term sleep quality, differentiating between sleep and wake to compute total sleep time, sleep efficiency, and instances of wake after sleep onset. WA devices are wireless, portable, and can be worn in a free-living environment. These devices infer sleep and wake via an accelerometer to detect the presence or absence of movement; however, they tend to have reduced sensitivity to wakefulness and thus inaccurately compute some metrics of overnight sleep quality, such as overestimating total sleep time^8–11^ and underestimating sleep onset latency (the time required for transition from wake to sleep).^12^ These metrics are used to compute an overall sleep efficiency, defined as the ratio of total sleep time to the amount of time in bed, with lower sleep efficiency corresponding to more time spent awake and poorer sleep quality. Importantly, the accuracy of actigraphy is reduced further for populations with an already low sleep efficiency, likely owing to the greater time spent awake by these populations and the reduced sensitivity to wake in WA devices.^11,13^ WA is often accepted in sleep research to objectively measure sleep in various healthy and patient populations, despite these limitations and without rigorous validation for individuals with limited upper limb mobility (e.g., stroke). Indeed, their dependence on acceleration-based movement alone suggest that they are unreliable to quantify sleep for populations with impaired or pathological movement patterns.^14^

In addition to the amount of movement, there are physiological mechanisms that change with wake and the different stages of sleep. These mechanisms reflect activity of the ANS,^15–18^ which regulates involuntary body functions such as respiration or heart rate. For example, distinct cardiovascular and thermophysical changes occur during sleep that is indicative of each sleep stage. Non-REM sleep is characterized by decreased heart rate, blood pressure, and blood flow to peripheral areas in the body, as well as an increased skin temperature and decreased core temperature. In contrast, REM sleep is characterized by fluctuating cardiovascular activity^18^ owing to modulations in sympathetic and parasympathetic system contributions in the ANS. Consequently, physiological changes during REM sleep include increased heart rate and less-efficient thermoregulation.^19–21^ Developing a system to accurately and continuously measure sleep architecture requires a fundamental trade-off between collecting enough relevant movement and physiological data to identify different sleep stages and ensuring that the system remains portable, ubiquitous, unobtrusive, and user-friendly.

State-of-the-art wireless, wearable sensors can adhere to the skin, flex around body contours, and collect multiple data modalities simultaneously. These devices enable continuous monitoring of health and disease states, including remote measurement of physical activity,^22^ vital signs,^23^ motor control symptoms of disease,^24^ and detection of falls.^25^ For specific applications in sleep monitoring, previous work has demonstrated automatic classification of sleep staging using multimodal sensor systems and machine learning, but many of these approaches still incorporate intrusive measures from the PSG^26–28^ or respiratory inductance plethysmography.^29,30^ Advanced wireless sensor technologies enable less-obtrusive access to physiological variables of interest, which may improve performance of machine learning classifiers that automatically identify sleep and sleep stages without negatively affecting sleep quality.

In this work, we propose a novel wireless and flexible sensor system that collects accelerometer, ECG, and skin temperature signals to determine sleep architecture with minimal intrusion. We applied machine learning techniques to classify sleep stages in healthy young adults, validated against PSG, and compared the performance of this system with WA and other state-of-the-art sleep classification using wireless sensors.

## Results

### Overnight sleep quality and gold standard sleep stage from PSG

Data from the proposed sensor set, the ActiWatch, and a PSG system were collected from a full night of sleep for 11 healthy young adults. A trained technician scored the PSG recordings as being wake (time from lights off until sleep onset, or scored awakenings during the night until time of lights on), NREM1 (stage 1 non-REM sleep), NREM2 (stage 2 non-REM sleep), SWS (slow wave sleep, stage 3 non-REM sleep), or REM sleep. NREM1 and NREM2 are considered light sleep, whereas SWS is considered deep sleep. Overnight sleep quality for the study participants is summarized in Table 1. Sensor data were labeled based on accompanying PSG sleep stage scores and then segmented into two-minute clips. Features were computed for each data clip and used to train population-based bagging decision tree classifiers. Performance of the bagging classifier was compared with that of the ActiWatch and to various alternative machine learning models described in the Supplementary Methods, Supplementary Table 1, and Supplementary Fig. 1. Personal models were also tested to evaluate the ability to classify sleep stages based on an individual’s own data.

**Table 1:** Participant characteristics and PSG sleep architecture measures.

Sleep quality metric	Mean (SD)
PSQI Global Score	3.7 (2.1)
Total sleep time (min)	425.75 (32.6)
Sleep efficiency (%)	88.9 (6.8)
Sleep onset latency (min)	15.1 (11.4)
Latency to persistent sleep (min)	25.8 (23.5)
WASO (min)	29.5 (23.7)
Stage 1 (%)	4.4 (1.8)
Stage 2 (%)	51.6 (8.6)
Stage SWS (%)	27.0 (7.7)
REM sleep (%)	16.9 (6.5)
REM latency (min)	194 (89.2)

*PSQI* Pittsburgh Sleep Quality Index, *WASO* wake after sleep onset, *SWS* slow wave sleep, *REM* rapid eye movement

### ActiWatch often misclassifies periods of wake as sleep

**Fig. 1:**
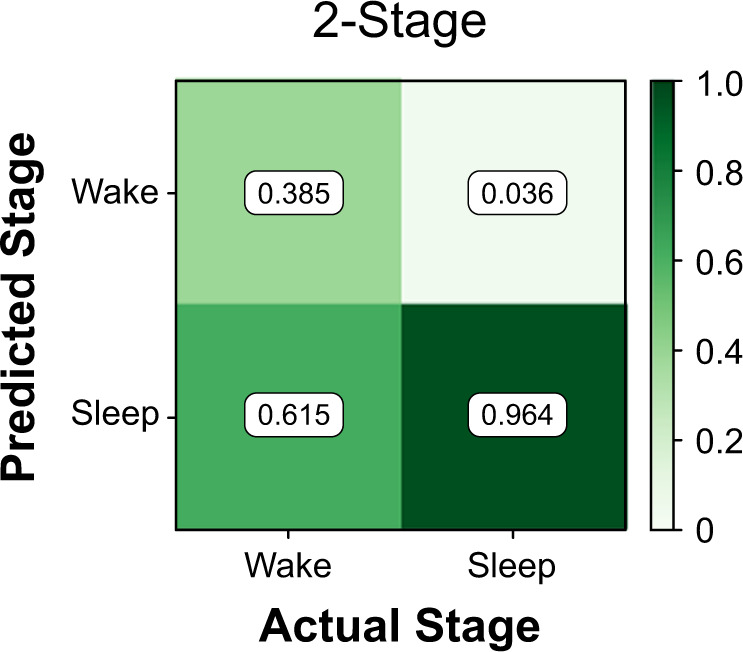
Performance of ActiWatch wrist sensor. Confusion matrix for the ActiWatch in a two-stage resolution, depicting average classification rate of wake and sleep stages. The ActiWatch demonstrated high recall of sleep (high sensitivity) but often misclassified Wake as sleep (low specificity).

The ActiWatch outputs an automated sleep vs. wake classification in 30-second clips. The classifications from ActiWatch were compared with the corresponding PSG label, thereby evaluating the reliability of the WA control device against the gold standard. Across participants, the ActiWatch was able to recall an average of 96.4 ± 0.88% of sleep epochs, but only 38.5 ± 19.2% of wake (Fig. 1).

### Proposed sensor system improves sleep stage classification

Bagging classifiers were built for three different resolutions of sleep staging:

- Two-stage wake vs. sleep (PSG stages NREM1, NREM2, SWS, REM).
- Three-stage wake vs. NREM sleep (PSG stages NREM1, NREM2, SWS) vs. REM sleep.
- Four-stage wake vs. light sleep (PSG stages NREM1, NREM2) vs. deep sleep (PSG stage SWS) vs. REM sleep.

**Fig. 2:**
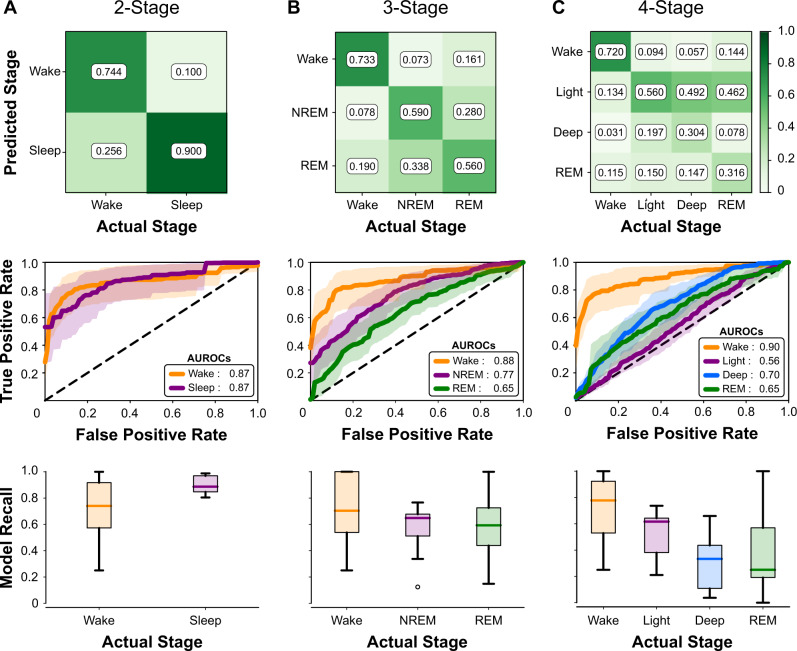
Performance of a bagging decision tree classifier for different sleep staging resolutions. Confusion matrices (top), Receiver Operating Characteristic (ROC) curves (middle), and interquartile range (IQR) plots of model performance (bottom), obtained from leave-one-out cross-validation subject, for **a** two-stage wake vs. sleep classification, **b** three-stage wake vs. NREM vs. REM classification, **c** four-stage wake vs. light vs. deep vs. REM classification. ROC curves show the trade-off between sensitivity and specificity for a given model across subjects (line: mean; shading: standard deviation). Area under the ROC curve (AUROC) is listed for each stage; a value of 1.0 denotes a perfect classifier, whereas a value of 0.5 denotes a classifier that performs no better than random and has no predictive power. IQR plots illustrate how well the model generalizes across subjects, with smaller ranges indicating good performance and high generalizability irrespective of the subject (center line: median; box limits: upper and lower quartiles; whiskers: 1.5 × IQR; points: outliers).

Performance of these classifiers is shown in Fig. 2. The two-stage model correctly identified wake and sleep for 74.4 ± 23.7% and 90.0 ± 7.1% of clips, respectively (Fig. 2a). For three-stage resolution (Fig. 2b), most confusion for the classifier was between NREM and REM sleep stages, while the classification performance of wake remained mostly intact. The classifier shows a moderate amount of predictive power for discerning whether sleep is REM or NREM, as shown by the area under the receiver operating characteristic (AUROC) values > 0.5. The four-stage resolution (Fig. 2c) performs relatively poorly, over-predicting the light sleep stage and showing low generalizability across subjects, but retains a wake recall similar to the three-stage model. This approach substantially outperforms the ActiWatch for predicting wake at all resolutions. The bagging model was the best-performing population model explored in this study (Supplementary Table 2).

Personal models developed from a similar bagging classifier approach notably improved performance for the four-stage resolution (Supplementary Fig. 2). On average, personal models correctly identified wake, light, deep, and REM sleep in 69.5%, 47.8%, 67.2%, and 57.9% of clips, respectively, with most confusion occurring between the light and deep stages.

### Effect of sensor location

To facilitate practical implementation of sleep monitoring with wearable sensors, we sought to minimize the number of sensors needed to maintain performance of the sleep stage classifier. Different location subsets of the proposed sensors were selected and tested for each model resolution using the bagging classifier. The subsets were chosen systematically to test the contribution of various sensors to overall classification performance and to group the sensors in as few body locations as possible for future device development. Each subset and its average AUROCs are provided in Table 2. The minimal sensor configuration required to maintain classifier performance in the two-, three-, and four-stage models was a single accelerometer (non-dominant), hand skin temperature (non-dominant), and ECG. The AUROCs for this minimal sensor set in the three-stage model to identify wake, NREM, and REM were 0.88 ± 0.15, 0.77 ± 0.14, and 0.65 ± 0.15, respectively, whereas the AUROCs for the full 13 sensor set was 0.90 ± 0.11, 0.74 ± 0.13, and 0.66 ± 0.19. All analyses are shown using this minimal sensor set to directly illustrate its utility.

**Table 2:** Mean (SD) AUROC for different subsets of the proposed sensor system for the two-, three-, and four-stage resolution models.

Sleep stage	ACC, ECG, TEMP (all)	ACC ND, ECG, all distal TEMP	ACC ND, ECG, all proximal TEMP	ACC ND, ECG, Chest TEMP (single proximal sensor), hand TEMP ND (single distal sensor)	ACC ND, ECG, hand TEMP ND	ACC ND, ECG	ACC ND	ECG	Hand TEMP ND
Wake (2-stage)	0.89 (0.12)	0.86 (0.16)	0.84 (0.15)	0.87 (0.16)	**0.87** **(0.16)**	0.85 (0.14)	0.82 (0.19)	0.72 (0.21)	0.71 (0.16)
Sleep (2-stage)	0.89 (0.12)	0.86 (0.16)	0.84 (0.15)	0.87 (0.16)	**0.87** **(0.16)**	0.85 (0.14)	0.82 (0.19)	0.72 (0.21)	0.71 (0.16)
Wake (3-stage)	0.90 (0.11)	0.87 (0.15)	0.84 (0.16)	0.87 (0.16)	**0.88** **(0.15)**	0.83 (0.17)	0.81 (0.18)	0.67 (0.24)	0.72 (0.17)
NREM (3-stage)	0.74 (0.13)	0.75 (0.13)	0.75 (0.12)	0.76 (0.13)	**0.77** **(0.14)**	0.76 (0.11)	0.68 (0.11)	0.71 (0.12)	0.58 (0.15)
REM (3-stage)	0.66 (0.19)	0.62 (0.17)	0.63 (0.16)	0.64 (0.15)	**0.65** **(0.15)**	0.65 (0.15)	0.45 (0.13)	0.68 (0.11)	0.50 (0.14)
Wake (4-stage)	0.90 (0.11)	0.87 (0.15)	0.85 (0.14)	0.88 (0.14)	**0.89** **(0.13)**	0.85 (0.14)	0.82 (0.18)	0.70 (0.24)	0.71 (0.17)
Light (4-stage)	0.53 (0.10)	0.57 (0.09)	0.56 (0.09)	0.58 (0.10)	**0.58** **(0.10)**	0.57 (0.10)	0.58 (0.05)	0.59 (0.09)	0.51 (0.08)
Deep (4-stage)	0.71 (0.10)	0.70 (0.09)	0.68 (0.09)	0.70 (0.09)	**0.71** **(0.09)**	0.70 (0.09)	0.64 (0.10)	0.65 (0.11)	0.57 (0.11)
REM (4-stage)	0.68 (0.15)	0.65 (0.14)	0.66 (0.14)	0.67 (0.13)	**0.67** **(0.14)**	0.68 (0.13)	0.47 (0.09)	0.70 (0.10)	0.49 (0.13)

*ACC* accelerometer, *ECG* electrocardiography, *TEMP* skin temperature, *ND* non-dominant side

The minimum sensor set is presented in bold

Removing skin temperature as a sensor modality reduced performance for detecting wake (AUROC 0.83 ± 0.17). Using only acceleration or only hand temperature decreased performance for detecting all three stages, whereas using only ECG decreased performance for detecting wake and NREM, but not REM.

### Effect of training sample size

**Fig. 3:**
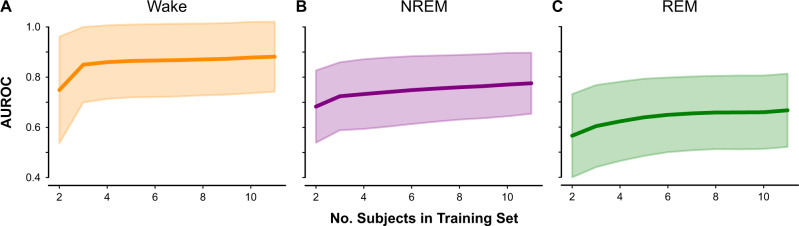
Effect of number of training subjects on model performance. Mean and standard deviation (shading) of AUROC for **a** wake, **b** NREM, and **c** REM classes in the three-stage bagging classifier model. The gradual increase in AUROC for each class suggests that a training set larger than *N* = 11 would continue to improve classification performance.

To estimate the performance impact of increasing the sample size, the data set was reduced to only two subjects. The data set size was incrementally increased by one until all subjects were again in the data set, evaluating the classifier AUROC and its standard deviation at each number of subjects (Fig. 3). This shows a performance increase as subjects are added, through an increase in AUROC and a decrease in the standard deviation of the AUROC. Performance does not appear to plateau as subjects are added back into the training set, indicating that the inclusion of more subjects will likely continue to improve performance.

### Comparison with other work

We compared our two-stage findings from the proposed sensor system and the ActiWatch to that of five previous studies using wireless, wearable sensors to automatically detect sleep and wake (Table 3). These studies have recorded accelerometry, gyroscope, ECG, plethysmography (PPG), actigraphy, and skin temperature data. Our approach yields the highest specificity (detection of wake) and a slightly lower, though comparable, sensitivity (detection of sleep).

**Table 3:** Comparison of current study results (proposed sensor set and ActiWatch) to previous wearable sensor work.

Study	Sensor modalities	Subjects	Model	Specificity (detection of wake)	Sensitivity (detection of sleep)
**Proposed System**	**ACC ND; ECG; Hand TEMP ND**	**11**	**Bagging classifier; 51 features extracted from 2** **min epochs**	**74.4%**	**90.0%**
Beattie et al.^31^	ACC; PPG	60	Linear discriminant classifier; 54 features extracted from 30 s epochs	69.3%	94.6%
Aktaruzzaman et al.^43^	ACT; ECG	18	Support vector machine classifier; 4 features extracted from 7 min epochs	54%	81%
De Zambotti et al.^44^	PPG; ACC; Gyroscope; TEMP	41	Proprietary algorithm by ŌURA Ring (Oulu, Finland); direct comparison between wake/sleep output and PSG for each 30 s epoch	48%	96%
Fonseca et al.^45^	ACC; PPG	152	Linear discriminant classifier; 54 features extracted from 30 s epochs	58.2%	96.9%
Razjouyan et al.^46^	ACC; ACT	21	Threshold optimized by accuracy rate for wake/sleep score; based on 1 min epochs	53.4%	94.9%
**ActiWatch**	**ACT**	**11**	**Built-in threshold for wake/sleep score; based on 30** **s epochs**	**38.5%**	**96.4%**

Bolded rows indicate results from the current study

Results from other recent wearable sensor studies examining higher resolution sleep staging or alternative formulations of stage discrimination are summarized in Supplementary Table 3. The best-performing four-stage model in recent work is from Beattie et al.,^31^ using a wrist-worn device collecting PPG and ACC with a linear discriminant classifier. This study obtained classification accuracies of wake: 69.3%, light: 69.2%, deep: 62.4%, and REM: 71.6%.

## Discussion

The sensor technology and machine learning approach employed in this study is a less invasive and lower cost method of sleep monitoring compared with the gold standard PSG system. A portable, automated system that can detect high-resolution sleep stages would significantly reduce the time for equipment setup and manual epoch scoring, as well as the financial costs for equipment, space, personnel, and training for overnight monitoring and subsequent scoring. Our minimal proposed sensor system—measuring accelerometer and skin temperature from the non-dominant hand, and ECG from the chest—outperformed the ActiWatch for classifying restful wake (proposed system 74.4% vs. ActiWatch 38.5%), with slight reduction in performance for classifying sleep (proposed system 90.0% vs. ActiWatch 96.6%). Our approach also yielded the best average performance for classifying wake compared with other studies of wireless sensors for sleep detection. Improved detection of wake would allow clinicians and researchers to compute more-accurate metrics of sleep quality from wireless body-worn technology, such as sleep onset latency, total sleep time, and occurrences of wake after sleep onset.

This approach also demonstrates potential for higher resolution sleep stage monitoring than a traditional wrist-worn actigraphy device. In a three-stage resolution model, the minimal proposed sensor system classified wake, NREM, and REM with 73.3%, 59.0%, and 56.0% recall, respectively, with positive predictive power for each stage (AUROCs > 0.5). Although a four-stage resolution model showed a lack of generalizability and a preference for the majority class (light sleep), personal models more effectively identified wake, light, deep, and REM sleep, with 69.5%, 47.8%, 67.2%, and 57.9% average recall. Additional training data for the population-based models would likely improve classification of the three-stage and four-stage resolution models for reliable sleep stage monitoring.

We found the minimum number of sensors to maintain classification performance was five (one tri-axial accelerometer, three ECG sensors, one skin temperature sensor), significantly reduced from 13 sensors. This minimal sensor set offers a middle ground between ActiWatch and PSG, exhibiting improved performance over the ActiWatch with notably less intrusion than the PSG. To further improve the feasibility of this system for long-term, portable, and user-friendly monitoring, different sensor modalities can be packaged into a single wrist- or hand-worn system, measuring acceleration, skin temperature, and replacing ECG with PPG. Also, provided that additional data would improve model accuracy, performance of the three- and four-stage classifiers could be improved to lessen the performance disparity between the PSG and the proposed sensor set.

Including skin temperature data generally improved classification of restful wake. Although core body temperature is more closely tied to sleep, circadian rhythms, and ANS regulation than skin temperature,^32^ current methods to measure core temperature are intrusive (i.e., rectal or vaginal thermometers). Nevertheless, the distal-to-proximal skin temperature gradient is a good predictor of sleep initiation,^33^ as an indirect measure of distal heat loss. Whereas proximal skin temperature follows the same circadian time-course as core temperature, decreasing to prepare for sleep and increasing to prepare for wake,^34^ distal skin temperature follows the reverse pattern and represents vasodilation and heat loss at distal regions of the body that promotes sleep onset.^35^ Although we computed distal-to-proximal temperature gradient (DPG) features when testing the full sensor set, distal temperature from a single sensor at the hand was sufficient to maintain model performance. A possible explanation is that the distal skin temperature is a more direct indicator of the drop in core body temperature, and the large temperature changes peripheral body that accompany sleep onset^35,36^ outweighed the contributions of proximal skin temperature during the machine learning process. We tested a distal sensor at the hand to consider whether this modality could be paired with accelerometer. Indeed, it improved classification of sleep and wake over accelerometer alone or accelerometer and ECG.

The primary limitation of this study is the small sample size. Traditional machine learning models and neural networks perform best when a large amount of training data are used to adequately learn the underlying data trends.^37^ Though the model held predictive power for detecting sleep and wake (two-stage resolution), classification of additional sleep stages (three- and four-stage resolution) was limited, likely owing to the more subtle physiological changes and intersubject variability that occurs in NREM and REM sleep. As shown in Fig. 2 (bottom panel), the range of AUROC for detecting sleep in the wake vs. sleep model is relatively low, meaning that this model performs consistently well to detect sleep irrespective of the subject. In contrast, the range for the higher resolution stages of sleep vary greatly, meaning that the model performs well to classify the various sleep stages for some subjects, but poorly for others. Personal models demonstrated improved classification performance at the four-stage resolution, suggesting that there are subject-specific differences in these stages that were not sufficiently captured by a population-based model. Increasing the subject sample size would expand the training data set, and likely improve differentiation for higher resolution sleep staging as well as generalization to new subjects.

Another limitation of the work is in the use of a single technician to score the PSG data. A previous study of the inter-rater reliability of PSG scoring by trained sleep technicians found 82.6% overall agreement for sleep stages, with disagreements between scorers primarily occurring in the transitions from one stage to another.^38^ We have reduced the potential for ambiguity in the PSG scores by removing transitions from the data (see Materials and methods, Signal alignment), but future studies should consider using multiple PSG scorers to ensure accuracy of the gold standard sleep stages.

Future work will gather additional training data to test existing and proposed models and apply this wireless sensor system to monitor sleep in patient populations, including individuals with stroke. Sleep stage cycling promotes neuroplasticity, which in turn can improve motor learning and structural recovery of damaged brain.^39^ This may be especially important in defining appropriate therapies to enable recovery after stroke or traumatic brain injury, where the main cause of disability is the damage to brain circuits. However, sleep is often suboptimal for these hospitalized patients, in part owing to disturbances during the night.^40^ Thus, regular monitoring of patient sleep may inform the recovery process and quantify impact of environment on sleep quality. As the proposed sensor set performed better than the ActiWatch for healthy individuals, and the ActiWatch has shown decreased accuracy in those with poor sleep efficiency,^13^ we believe the proposed set is a more effective solution for wireless monitoring. It remains to be seen how this sensor data from an impaired population would affect the model training and performance.

An additional area of interest for future work is to continue exploring alternative machine learning approaches to optimize classification accuracy. For example, previous algorithms used for sleep classification (but with different sensor signals) can be applied to the same data set to directly compare model performance. We found that a simple population-based bagging classifier performed well on average for this small data set, but we expect that more sophisticated learning algorithms, such as sequence-based classifiers or recurrent neural networks, would be effective learning algorithms in this problem with more training data.

## Methods

### Participants

Twelve individuals (7F/5M, age 27.4 ± 3.9 years, BMI 24.2 ± 2.7 kg/m^2^) participated in the study after providing written informed consent and meeting the following inclusion criteria: (1) older than 18 years of age; (2) independent in activities of daily living; (3) able to give a written informed consent; (4) no history of significant medical, neurological, or psychiatric illness. Exclusion criteria included: (1) subjective report of sleep disorders by history or documented on PSG, (2) shift work or other types of self-imposed irregular sleep schedules; (3) body mass index > 35 kg/m^2^. Data from one participant was excluded owing to a sensor malfunction, leaving data from 11 participants to be used for the training and testing of machine learning models.

### Study protocol

The protocol was approved by the Northwestern University Institutional Review Board. Eligible participants came to the Northwestern Memorial Hospital Sleep Disorders Center for one night of PSG sleep and multimodal sensor monitoring. Participant bedtime was determined using the self-reported habitual bedtime of each patient. Data were collected from the time of lights off to the time of lights on, 8 h later, using the Polysmith v8.0 software (Nihon-Kohden; Gainesville, FL). Participants completed a Pittsburgh Sleep Quality Index (PSQI) self-reported sleep questionnaire prior to sleep monitoring setup.^41^

**Fig. 4:**
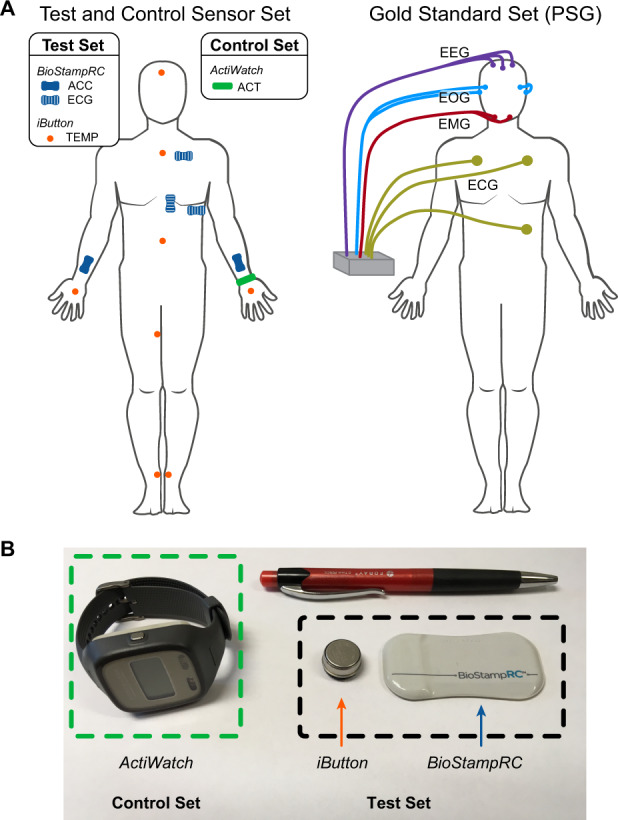
Sensor systems and placement during overnight sleep. **a** Each participant wore three systems, including the proposed sensor set, consisting of accelerometers (ACC), electrocardiography (ECG), and skin temperature (TEMP), in addition to the wrist actigraphy control device measuring activity counts (ACT) and the gold standard system (PSG). **b** Size and profile comparison of the proposed sensors with the control device. (iButton: Copyright Maxim Integrated Products. Used by permission. ActiWatch: Permission to use ActiWatch Spectrum image was granted by Philips Respironics. BioStampRC: Permission to use BioStampRC image was granted by MC10, Inc.).

Participants concurrently wore three different sensor systems: (1) the proposed set, consisting of the accelerometer, ECG, and skin temperature sensors, (2) the control set, consisting of a research-grade WA device, and (3) the gold standard PSG system (Fig. 4a). To place the sensors, the skin was prepped with alcohol and electrode gel was added to ECG sensors. Additional adhesive dressings (Tegaderm; 3M, Maplewood, MN, USA) were applied over the proposed sensor set devices to stabilize the device-skin interface. Trained sleep technicians and researchers placed all sleep monitoring systems on participants prior to lights off.

### Sleep monitoring systems

The proposed wearable sensor set was composed of the BioStampRC (MC10, Inc.; Lexington, MA) and the Thermochron iButton (DS1922L‐F5; Maxim Integrated Products, San Jose, CA, USA). These devices are both wireless and have a low profile, and the BioStampRC is a flexible sensor with a soft-shell encasing (Fig. 4b); such characteristics are beneficial to maximize participant comfort over the wired and cumbersome PSG system. Two BioStampRC sensors, placed at the same orientation, collected tri-axial acceleration bilaterally at the wrist (sampling frequency of 62.5 Hz). Three BioStampRC sensors collected ECG at the chest (sampling frequency of 1000 Hz). Although these are single-lead ECG devices, we placed three sensors to create redundancy in the event of noisy signals or movement artifact from any one sensor throughout the night. Eight iButton sensors collected skin temperature (sampling frequency of 1/60 Hz), with four placed distally at the back of each hand and below the medial malleolus of each ankle, and four placed proximally at the forehead, chest, abdomen, and right inner thigh in accordance with placement described in Krauchi et al.^35^ Data from the BioStampRC and iButton sensors were stored on the device and later offloaded for analysis.

The ActiWatch Spectrum (Philips Respironics; Murrysville, PA) served as the WA control device. The ActiWatch contains a tri-axial piezoelectric accelerometer and records an activity count for each 30-second epoch by summing peak acceleration per second over the length of the epoch. This activity count determines sleep or wake classification based on a built-in thresholding algorithm. Participants wore the ActiWatch on their non-dominant wrist.

The gold standard PSG system included four scalp electrodes for monitoring EEG, at central (C3, C4) and occipital (O1, O2) locations and reference electrodes on the contralateral mastoid (A1, A2). Electro-oculogram (EOG), electromyogram (EMG), and electrocardiogram (ECG) were also obtained. Signals were amplified and sampled at 200 Hz (Neurofax EEG-1100, Nihon-Kohden), with a 70 Hz low-pass filter and a time constant of 0.3 s (0.6 Hz). A Registered Polysomnographic Technologist scored each 30-second epoch of the recording using the Polysmith software as wake or one of four sleep stages, according to the American Academy of Sleep Medicine (AASM) scoring criterion. These sleep stages included stage 1 (NREM1), stage 2 (NREM2), slow wave sleep (SWS), and REM sleep.^6^ Sleep metrics computed from the PSG scores include the following:

- Total sleep time: time interval separating sleep onset latency from morning awakening minus the amount of time spent awake during the night.
- Sleep efficiency: total sleep time divided by the total recording time and expressed as a percentage.
- Sleep onset latency: time from lights off to the first 30-second epoch scored as sleep stage 1 or higher.
- Latency to persistent sleep: time from lights off to the time the onset of sleep lasting at least 10 contiguous minutes in any sleep stage.
- Wake after sleep onset (WASO): time spent awake after sleep onset and before lights on.
- Percentage of each sleep stage: ratio of time spent in each sleep stage to the total sleep time.

### Data cleaning

All preprocessing was performed in MATLAB 2018b software (Natick, MA, USA). Data from the BioStampRC sensors were interpolated and resampled to 62.5 Hz for accelerometer data and 1000 Hz for ECG data, to correct for occasional duplicated or missing data points. Initial noise in the BioStampRC ECG signal was mitigated using the following approach: the standard deviation of the signal was computed over 10-second clips. Any clip with a standard deviation higher than twice the mean of the lowest 15% of standard deviations was not included for analysis. Individual outliers, identified as data points > 10 standard deviations above the mean, were removed from the clip as well as a 5 ms buffer before and after these outlier points. Signals of the three ECG sensors then were summed to create a composite ECG signal.

### Signal alignment

**Fig. 5:**
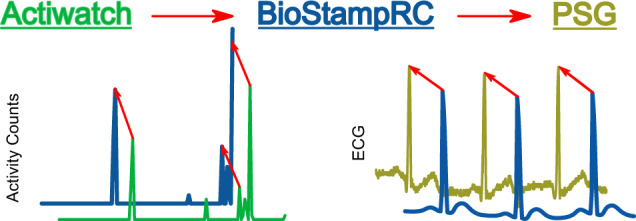
Time synchronization of independent data collection systems. ActiWatch was synchronized with the BioStampRC by aligning activity counts; BioStampRC was synchronized to the PSG by aligning ECG signals.

Because the PSG, ActiWatch, and proposed sensor set were not part of a single data acquisition system, the time-series data were not automatically synchronized between systems. Synchronization is vital to ensure that the training data used in machine learning algorithms are labeled correctly to match the gold standard sleep stage from the PSG. ActiWatch and BioStampRC data were synchronized post hoc with the PSG data using the MATLAB function *xcorr*, which maximizes the cross-correlation between two signals by shifting one of the signals in time. Our strategy for time-series alignment is depicted in Fig. 5, where the ActiWatch is first synchronized to the BioStampRC by aligning wrist acceleration signals (after transforming the BioStampRC acceleration into an approximate activity count via the method in Te Lindert et al.^42^), and the BioStampRC is synchronized to the PSG by aligning ECG signals. The iButtons required no alignment correction because they were initialized on the PSG computer system, and so were already time-synchronized with the PSG. Alignment of all systems was confirmed via visual inspection.

The PSG score provided a gold standard sleep stage to the aligned sensor and ActiWatch data. The 30-second epochs immediately before and after a sleep stage transition were removed, as PSG epochs are scored based on the majority stage, but it is unclear from the score when the transition from one stage to another occurs. We removed these potential transitional clips to ensure that the sensor data were fully consistent with the aligned PSG score and thereby maintain the integrity of training data for the machine learning models.

### Signal processing

ECG and accelerometer data from the BioStampRC were high-pass filtered using a Butterworth filter with a cutoff frequency of 1 Hz and an order of 1st and 5th, respectively, to remove signal drift. The wavelet transform of the ECG data was computed using the “sym4” wavelet, which resembles the QRS complex of the ECG trace and accentuated the R-peaks in the signal. The time locations of R-peaks in the ECG trace were determined using the MATLAB function findpeaks on the transformed signal, which were then used to calculate R-R intervals to compute measures of heart rate and heart rate variability.

### Feature extraction

Sensor data were segmented into non-overlapping 2-minute clips, each with a corresponding PSG score as the true sleep stage. This resulted in 10,527 total clips available for machine learning models, 45% of which were from the NREM2 stage (Supplementary Fig. 3). Fifty-one features were computed for each clip, including 33 from the accelerometer (11 per axis) in the time domain, 14 from the ECG in both the time and frequency domain, and 4 from skin temperature in the time domain. These features are listed in Table 4.

**Table 4:** Features extracted from the sensor data.

Sensor modality	Sampling frequency (Hz)	No. of features	Features
Accelerometer	62.5	33	Mean (x,y,z) Minimum (x,y,z) Maximum (x,y,z) Range (x,y,z) Interquartile range (x,y,z) Standard deviation (x,y,z) Kurtosis (x,y,z) Root mean squared (x,y,z) Variance (x,y,z) Pearson’s coefficient (x,y,z) Pearson’s *p* value (x,y,z)
ECG	1000	14	Mean R-R interval Minimum R-R interval Maximum R-R interval Standard deviation R-R interval RMSSD NN50, PNN50 NN20, PNN20 VLF, LF, HF LF/HF Ratio
Skin temperature	0.0167	4	Mean DPG Minimum DPG Maximum DPG Range DPG

*RMSSD* root mean square of successive differences; NN*X* number of successive R-R intervals that differ by more than *X* ms, PNN*X* ratio of NN*X* to total number of R-R intervals, *VLF* very low frequency power (activity in the 0.003–0.04 Hz frequency band); *LF* low frequency power (activity in the 0.04–0.15 Hz frequency band), *HF* high frequency power (activity in the 0.15–0.40 Hz frequency band); *DPG* distal-to-proximal gradient^35^

### Sleep classification

A bagging classifier with a decision tree estimator was used for supervised machine learning. This ensemble learning approach is advantageous for its resistance to overfitting and small number of tunable hyperparameters. An ensemble of 130 decision tree classifiers was trained using a random subset from the feature matrix. This number of trees was sufficient to achieve nearly full learning without overfitting the model. To account for the imbalance of sleep stage classes, the bagging classifier was coupled with random under-sampling to reduce preference for predicting the majority class (NREM2).

We also explored various alternative machine learning approaches for the classification of sleep stages, including a Support Vector Machine, Convolutional Neural Network, Hidden Markov Model, and Long Short-Term Memory model. However, none of these models outperformed the ensemble-based bagging classifier, so we focus predominantly on results from the bagging classifier in the main text. The alternative models and their formulation are described in the Supplementary Methods.

The models were developed to classify sleep stage for three different resolutions of sleep staging: the two-stage wake vs. sleep (PSG stages 1, 2, SWS, REM); the three-stage wake vs. NREM sleep (PSG stages 1, 2, SWS) vs. REM sleep; and the 4-stage wake vs. light sleep (PSG stages 1, 2) vs. deep sleep (PSG stage SWS) vs. REM sleep.

Model performance was evaluated as via the model recall (true positive rate, also known as sensitivity in a two-class problem) and AUROC, which were computed using leave-one-subject-out cross-validation. In this population-based approach, data from other subjects are used to detect sleep stages for a new subject.

The bagging model was trained and tested using data from targeted subsets of the proposed sensors to minimize the total number of sensors required while maintaining classification performance. The sensor subsets were compared using AUROC for each sleep stage, which provides a single measure of the separability of that sleep stage from the others. For subsets of the skin temperature sensors, features were computed on the DPG^35^ when using all temperature sensors, on the weighted temperature average when using either all distal or proximal sensors, or on the pure temperature when using individual sensor locations.

In addition, we implemented personal models for each subject, wherein data from one subject were used to detect sleep stages in the same subject. Balanced bagging classifiers with 130 trees were tested using 20-fold cross-validation for each subject, then averaged across subjects. This analysis was introduced to address the potential individual differences in sensor data for this relatively small data set, which may not generalize well for a population-based approach.

### Comparison with other work

A literature search was conducted to compare our results with previous work in wearable sensor sleep classification using the keywords “sensors,” “sleep detection,” “sleep classification,” and “machine learning.” Studies were included for direct comparison if they met the following criteria: (1) trained machine learning models on PSG data from wireless, wearable technology, and (2) reported model recall for sleep vs. wake classification or for multiple sleep stages using non-hierarchical methods. Studies using wired or intrusive sensors (i.e., wired EEG, rectal core temperature) were excluded.

### Reporting summary

Further information on experimental design is available in the Nature Research Reporting Summary linked to this paper.

## Supplementary information

npj Reporting Summary

Supplementary Material

## Data Availability

The training data set may be made available to an investigator upon request for academic, research, and non-commercial use, subject to any license.

## Code availability

The code used to process and analyze the findings of this publication may be made available to an investigator upon request for academic, research, and non-commercial use.
