# Supplementary material for: Automating sleep stage classification using wireless, wearable sensors
Source: NPJ Digit Med. 2019 Dec 20;2:131. doi: 10.1038/s41746-019-0210-1 (PMC6925191; doi:10.1038/s41746-019-0210-1)
Supplement: Supplementary file 2 — Supplementary Material [file 41746_2019_210_MOESM2_ESM.pdf]

SUPPLEMENTARY MATERIAL

Automating sleep stage classification using wireless, wearable sensors

Authors: Alexander J. Boe[1,2*], Lori McGee Koch[1,3*], Megan K. O'Brien[1,4], Nicholas Shawen[1,5], John A. Rogers[6], Richard L. Lieber[2,4,7], Kathryn J. Reid[3], Phyllis C. Zee[3], Arun Jayaraman[1,4†]

* *These authors should be considered co-first authors*
† *Corresponding Author*

[1] Max Nader Lab for Rehabilitation Technologies and Outcomes Research,
Shirley Ryan AbilityLab, Chicago, IL 60611, USA

[2] Department of Biomedical Engineering,
Northwestern University, Evanston, IL 60208, USA

[3] Department of Neurology,
Northwestern University, Chicago, IL 60611, USA

[4] Department of Physical Medicine and Rehabilitation,
Northwestern University, Chicago, IL 60611, USA

[5] Medical Scientist Training Program,
Northwestern University Feinberg School of Medicine, Chicago, IL 60611, USA

[6] Center for Bio-Integrated Electronics, Departments of Materials Science and Engineering, Biomedical Engineering, Electrical Engineering and Computer Science, Northwestern University, Evanston, IL 60208, USA

[7] Shirley Ryan AbilityLab, Chicago, IL 60611, USA

Contact: ajayaraman@sralab.org

Supplementary Methods

In addition to the bagging classifier, we explored various alternative machine learning approaches for the classification of sleep stages, including hyperplane separation, deep learning, and probabilistic, sequence-based classification. These models and their formulation are as follows:

- *Support Vector Machine (SVM)*: SVMs are often used for classifying multi-dimensional data and while providing resistance to overfitting. These models can efficiently perform linear or non-linear hyperplane separation. For sleep stage classification, we tuned hyperparameters of a traditional SVM using leave-one-out cross validation. These parameters included C (soft margin constant), γ (kernel coefficient), kernel type (radial basis function [RBF] or polynomial [poly]), and degree (degree for polynomial kernel). To obtain initial estimates of the parameters, various sets were tested in a nested loop to find an optimal combination (C = [0.0001, 0.001, 0.01, 0.1, 1]; γ = [0.0001, 0.001, 0.01, 0.1, 1]; kernel = [RBF, poly]; degree = [2,3,4,5,6] for polynomial kernel only). Initial estimates were chosen as the parameters that maximized average accuracy from the cross validation. Final parameters were selected by repeating this process to fine-tune the initial estimates and are given in Supplementary Table 1.

- *Convolutional Neural Network (CNN)*: As a deep learning approach, the CNN does not require engineered features but rather learns from the signal data itself. Recently, neural networks have been used to automatically score data obtained from the PSG, with accuracies approaching that of a trained technician[1]. The CNN was trained using the

filtered sensor data instead of the pre-computed signal features used for the bagging classifier. The CNN consisted of pairs of convolutional layers and max pooling layers, followed by pairs of fully connected layers and dropout layers, before a final fully connected layer providing the output staging classification probabilities (Supplementary Figure 1). This architecture was adapted from previous work using similar sensor data to classify motor symptoms in Parkinson's disease[2].

We used a randomized bootstrapping method to tune and select the CNN hyperparameters, including the filter and kernel size for the convolutional layers, the number of neurons for fully connected layers, the pool size of the max pooling layers, and the batch size. Class weights were fixed as inversely proportional to the number of instances, such that the majority class was weighted as 1.0, and classes with fewer instances were weighted more heavily. In the parameter tuning process, model performance was computed using a random selection of the hyperparameters and a random subset of the subjects. Five subjects were used in each subset, with four subjects in the training set and the fifth subject to test the network. For each random set of parameters, five different subsets of subjects were tested, with the model performance metrics (validation loss, validation accuracy, and balanced accuracy) averaged over each random set. Final network parameters were selected after testing 500 random sets, as those that maximized the average balanced accuracy and minimized the average validation loss. The final tuned parameters for each resolution of the CNN are given in Supplementary Table 1.

- *Sequence-Based Classifiers*: We also used this dataset with sequence-based classifiers, specifically a Hidden Markov Model (HMM) and a Long Short-Term Model (LSTM). We expected sequence-based classifiers to be suitable for the challenge of sleep classification due to the cyclical nature of sleep stages. The HMM was trained using the 20 most important features, as determined from a random forest classifier. PSG sleep stage scores from all subjects were used to pre-compute the transition matrix and start probability for the HMM, while the covariance and average value matrices were computed from the sensor features at each sleep stage. The LSTM was trained using three time-distributed convolutional layers for feature extraction, followed by three LSTM layers. This architecture was adapted from a previous study by Bresch *et al.*[3] classifying sleep using a single EEG sensor. The convolutional layers for processing of ECG signals followed the structure used by these authors for the initial processing of EEG sensor data. We concatenated the ECG outputs with flattened outputs of time-distributed versions of the same accelerometer and temperature data-processing layers used in our CNN. This vector was then input into a series of LSTM layers, again following the structure in Bresch *et al*. The final parameters for each resolution of the LSTM are given in Supplementary Table 1.

Supplementary Figures

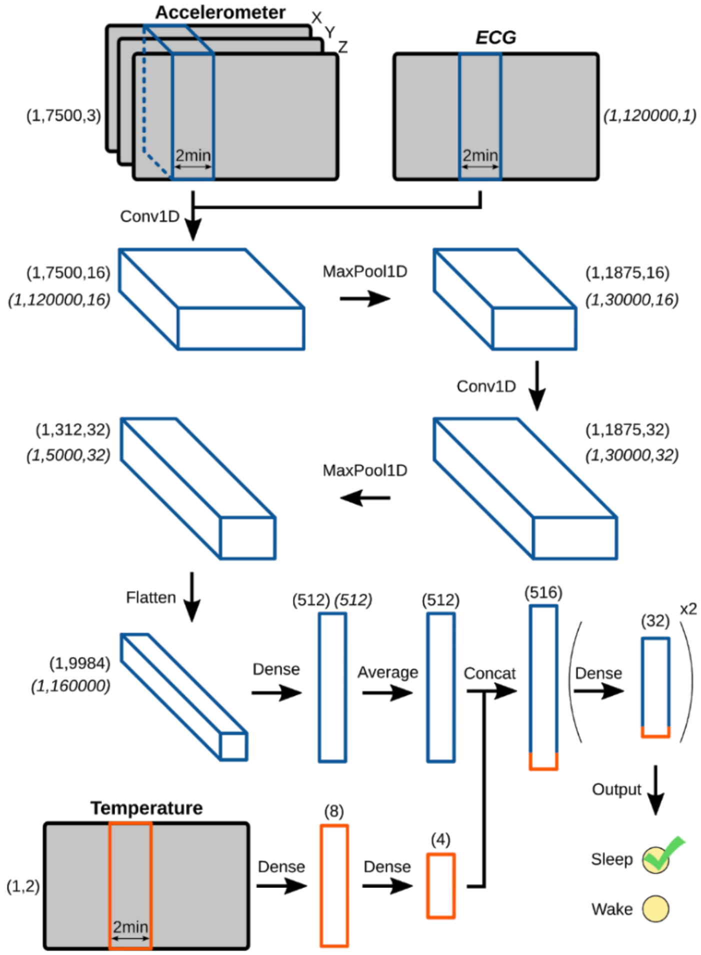

Supplementary Figure 1: Convolutional neural network architecture. The neural network incorporates raw data from the 3 sensor modalities in the proposed sensor system. The accelerometer and ECG data follow the same architecture in parallel to each other. The skin temperature data, with a notably lower sampling frequency, was incorporated downstream of the accelerometer and ECG data. The architecture starts with a series of convolutions with a filter size of 16 and a kernel of 32 for the first, and a filter size of 32 and a kernel of 16 for the second. Each convolutional layer is followed by a max pooling layer, with pool sizes of 4 and 6, respectively. The outputs of this are then flattened to prepare for the fully connected layers. First, the accelerometer and ECG are passed through a dense fully connected layer with 512 neurons and a rectified linear unit (ReLU) activation function. The two paths are then combined with an averaging merge layer, resulting in one output of size 512. The temperature data passes through 2 fully connected layers with 8 then 4 neurons, resulting in an output of size 4. The temperature and accelerometer/ECG tensors are then concatenated, resulting in an output of size 516. This is then passed through 2 more dense fully connected layers with 32 neurons and a ReLU activation function, each followed by a dropout layer to combat overfitting, wherein a specified proportion (0.5 in this case) of the neurons are silenced. The final layer is a softmax activation function dense fully connected layer with number of neurons matching the number of sleep stages, giving a final prediction of sleep stage.

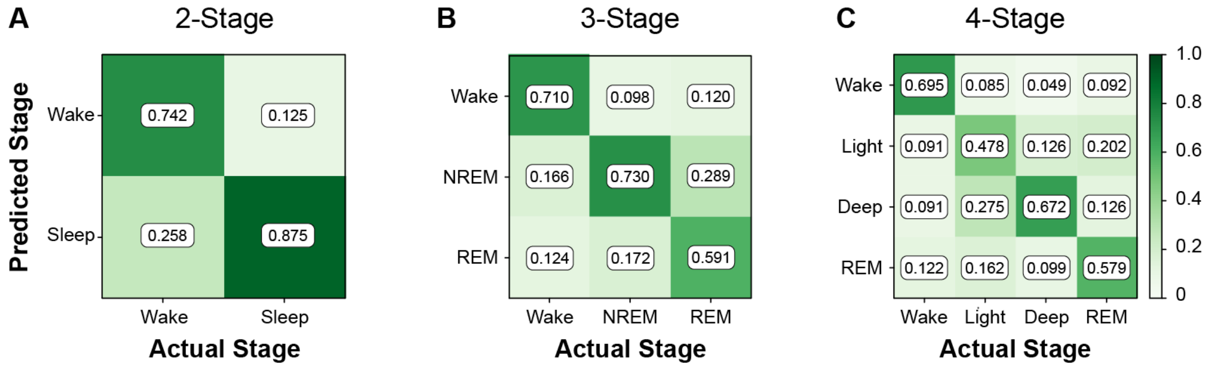

Supplementary Figure 2: Average performance of personal models. Confusion matrices obtained from bagging classifiers (130 trees) trained and tested on each subject separately via 20-fold cross validation, then averaged across subjects. Personal models exhibit a marked improvement in class recall over population models at the 4-stage resolution.

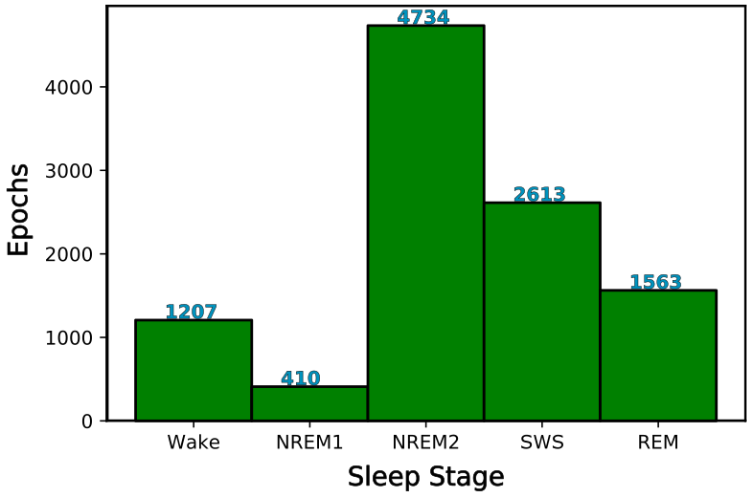

Supplementary Figure 3: Imbalance of sleep stages for algorithm training. Number of 30-second clips for each sleep stage contained in the dataset for model training and testing (10,067 clips total). NREM1 = Stage 1 sleep; NREM2 = Stage 2 sleep; SWS = Slow Wave Sleep (Stage 3 and 4 sleep); REM = Rapid Eye Movement.

Supplementary Tables

Supplementary Table 1. Hyperparameters for Support Vector Machine (CNN) and Convolutional Neural Network (CNN) models.

Model	Method	2-stage	3-stage	4-stage
SVM	Tuned<br><br>(Leave-One-Out Cross Validation)	C = 0.069<br>γ = 0.055<br>kernel = RBF	C = 0.057<br>γ = 0.05<br>kernel = RBF	C = 0.048<br>γ = 0.057<br>kernel = RBF
CNN	Selected<br><br>(Inversely proportional to number of instances in class)	<u>Class weights</u><br>w_{Wake} = 7.5<br>w_{Sleep} = 1.0	<u>Class weights</u><br>w_{Wake} = 6.4<br>w_{NREM} = 1.0<br>w_{REM} = 5.7	<u>Class weights</u><br>w_{Wake} = 4.2<br>w_{Light} = 1.0<br>w_{Deep} = 2.0<br>w_{REM} = 3.7
	Tuned<br><br>(Random bootstrapping)	• Filter size 1 = 2<br>• Kernel size 1 = 8<br>• Filter size 2 = 8<br>• Kernel size 2 = 16<br>• No. neurons = 16<br>• Pool size 1 = 16<br>• Pool size 2 = 8<br>• Batch size = 10	• Filter size 1 = 2<br>• Kernel size 1 = 16<br>• Filter size 2 = 32<br>• Kernel size 2 = 16<br>• No. neurons = 32<br>• Pool size 1 = 2<br>• Pool size 2 = 8<br>• Batch size = 20	• Filter size 1 = 4<br>• Kernel size 1 = 8<br>• Filter size 2 = 4<br>• Kernel size 2 = 4<br>• No. neurons = 128<br>• Pool size 1 = 4<br>• Pool size 2 = 16<br>• Batch size = 40
LSTM	Selected<br><br>(Adapted from previous publication[1])	• Filter size 1 = 8<br>• Kernel size 1 = 8<br>• Filter size 2 = 16<br>• Kernel size 2 = 8<br>• Filter size 3 = 32<br>• Kernel size 3 = 8<br>• Pool size (1,2,3) = 8<br>• LSTM 1 = 64<br>• LSTM 2 = 64<br>• LSTM 3 = 2	• Filter size 1 = 8<br>• Kernel size 1 = 8<br>• Filter size 2 = 16<br>• Kernel size 2 = 8<br>• Filter size 3 = 32<br>• Kernel size 3 = 8<br>• Pool size (1,2,3) = 8<br>• LSTM 1 = 64<br>• LSTM 2 = 64<br>• LSTM 3 = 4	• Filter size 1 = 8<br>• Kernel size 1 = 8<br>• Filter size 2 = 16<br>• Kernel size 2 = 8<br>• Filter size 3 = 32<br>• Kernel size 3 = 8<br>• Pool size (1,2,3) = 8<br>• LSTM 1 = 64<br>• LSTM 2 = 64<br>• LSTM 3 = 4

C: penalty of error term; γ: kernel coefficient; RBF: radial basis function; w_i: weight for class i

Supplementary Table 2. Performance comparison of population-based machine learning models, with average balanced accuracy and 95% confidence intervals.

Model	2-stage	3-stage	4-stage
Random class selection	0.50	0.33	0.25
Bagging	0.82 [0.75-0.90]	0.62 [0.55-0.70]	0.47 [0.43-0.52]
SVM	0.76 [0.70-0.83]	0.56 [0.50-0.63]	0.45 [0.39-0.51]
CNN	0.68 [0.59-0.78]	0.42 [0.37-0.47]	0.27 [0.24-0.31]
HMM	0.45 [0.42-0.48]	0.35 [0.26-0.44]	0.21 [0.17-0.25]
LSTM	0.50*	0.33*	0.25*

* The LSTM exclusively predicted sleep stages to be that of the previous class (did not deviate from the original Wake stage).

Supplementary Table 3. Additional wearable sensor studies not included in comparison to proposed system performance.

Study	Sensor Modalities	Subjects	Sleep Stage Resolution	Models	Best accuracy
Yuda et al. (2017)[4]	ACT; ECG	289	• REM. vs. Wake<br>• NREM vs. REM/Wake	Multivariate logistic regression	• REM. vs. Wake: 74.5%<br>• NREM vs. REM/Wake: 75.8%
Sano et al. (2014)[5]	ACC; TEMP; Skin Conductance	15	Wake vs. Sleep	SVM, k-NN	74% (including EEG features increases accuracy to 85%)*
Singh et al. (2016)[6]	ECG	20	REM vs. NREM	SVM	76.3%
Yeo et al. (2017)[7]	ACC	36	• Wake<br>• Light (NREM1+NREM2)<br>• Deep (NREM3)<br>• REM	K Star, Bagging, Random committee, Random subspace, Random forest	• Wake: >90%<br>• Light: >80%<br>• Deep: >90%<br>• REM: >90%
Ebrahimi et al. (2015)[8]	ECG	30	Wake vs. Light vs. Deep vs. REM	SVM with recursive feature elimination	89.32%
De Zambotti et al. (2019)[9]	PPG; ACC; Gyroscope; TEMP	41	Wake vs. Light (NREM1) vs. Deep (NREM2+NREM3) vs. REM	Proprietary algorithm by ŌURA Ring (Oulu, Finland)	• Wake: 48%<br>• Light: 65%<br>• Deep: 51%<br>• REM: 61%
Beattie et al. (2017)[10]	PPG; ACC	60	Wake vs. Light (NREM1+NREM2) vs. Deep (NREM3) vs. REM	Linear discriminant classifier, Quadratic discriminant classifier, Random forest, SVM	• Wake: 69.3%<br>• Light: 69.2%<br>• Deep: 62.4%<br>• REM: 71.6%

* Sensitivity/Specificity not reported for inclusion in Table 3 of the main text.

ACT = actigraphy; ECG = electrocardiography; ACC = accelerometry; TEMP = Skin temperature; PPG = plethysmography; SVM = support vector machine; k-NN = k-nearest neighbor.

Supplementary References

1. Biswal, S. *et al.* SLEEPNET: automated sleep staging system via deep learning. Preprint at https://arxiv.org/abs/1707.08262 (2017).

2. Lonini, L. *et al.* Wearable sensors for Parkinson's disease: Which data are worth collecting for training symptom detection models. *NPJ Digital Medicine* **1** (2018).

3. Bresch, E., Grossekathofer, U., & Garcia-Molina, G. Recurrent deep neural networks for real-time sleep stage classification from single channel EEG. *Frontiers in Computational Neuroscience*, 12, 85 (2018).

4. Yuda, E., Yoshida, Y., Sasanabe, R., Tanaka, H., Shiomi, T., & Hayano, J. Sleep stage classification by a combination of actigraphic and heart rate signals. *Journal of Low Power Electronics and Applications* **7,** 28 (2017).

5. Sano, A. & Picard, R. W. Comparison of sleep-wake classification using electroencephalogram and wrist-worn multi-modal sensor data. *36th Annual International Conference of the IEEE Engineering in Medicine and Biology Society*, 930-933 (2014).

6. Singh, J., Sharma, R. K., & Gupta, A. K. A method of REM-NREM sleep distinction using ECG signal for unobtrusive personal monitoring. *Computers in Biology and Medicine* **78,** 138-143 (2016).

7. Yeo, M., Koo, Y. S., & Park, C. Automatic detection of sleep stages based on accelerometer signals from a wristband. *IEIE Transactions on Smart Processing and Computing* **6,** 21-26 (2017).

8. Ebrahimi, F., Setarehdan, S-K., & Nazeran, H. Automatic sleep staging by simultaneous analysis of ECG and respiratory signals in long epochs. *Biomedical Signal Processing and Control* **18,** 69-79 (2015).

9. De Zambotti, M., Rosas, L., Colrain, I. M. & Baker, F. C. The sleep of the ring: comparison of the OURA sleep tracker against polysomnography. *Behavioral Sleep Medicine* **17,** 124–136 (2019).

10. Beattie, Z. *et al.* Estimation of sleep stages in a healthy adult population from optical plethysmography and accelerometer signals. *Physiological Measurement* **38,** 1968 (2017).
